# Long-term survivors demonstrate superior quality of life after haploidentical stem cell transplantation to matched sibling donor transplantation

**DOI:** 10.1186/s12967-022-03803-y

**Published:** 2022-12-14

**Authors:** Xiaoyu Zhang, Jiao Wang, Yuqiu Liu, Jie Liu, Bei Wang, Qiuhui Zhang, Wei Guan, Huijuan Zhang, Li Xu, Guiying Liu, Ping Zhang, Yi He, Sizhou Feng, Mingzhe Han, Changping Li, Erlie Jiang, Wenjun Xie

**Affiliations:** 1 Department of Hematopoietic Stem Cell Transplantation, State Key Laboratory of Experimental Hematology, National Clinical Research Center for Blood Diseases, Haihe Laboratory of Cell Ecosystem, Institute of Hematology & Blood Diseases Hospital, Chinese Academy of Medical Sciences and Peking Union Medical College, Tianjin, 300020 China; 2 Department of Public Health, Tianjin Medical University, 288 Nanjing Road, Tianjin, 300052 China; 3 Department of Nursing Care, State Key Laboratory of Experimental Hematology, Research Center for Blood Diseases, Haihe Laboratory of Cell Ecosystem, Institute of Hematology and Blood Diseases Hospital, National Clinical , Chinese Academy of Medical Sciences and Peking Union Medical College, 288 Nanjing Roa, Tianjin, 300020 China; 4 Clinical Research Division, Fred Hutchinson Cancer Center, Seattle, WA 98109 USA

**Keywords:** Allo-HSCT, HID-HSCT, Quality of life, aGVHD, CMV

## Abstract

**Background:**

It has been well-documented that haplo-identical hematopoietic stem cell transplantation (HID-HSCT) can provide outcomes comparable to conventional matched sibling donor (MSD) HSCT, however, little is known about the effects on quality of life (QoL) in long-term survivors. This study is to investigate the differences in longitudinal performance of QoL between HID and MSD HSCT using a comprehensive assessment system.

**Methods:**

This prospective study enrolled consecutive patients who had received allogenic-HSCT (allo-HSCT) between January 2018 and December 2019 in our center. All patients were informed to complete QoL questionnaires including the Mos 36-Item Short-Form Health Survey (SF-36) and the Functional Assessment of Cancer Therapy Bone Marrow Transplant (FACT-BMT, version 4), using an online applet, before transplantation and at scheduled time points after transplantation. The linear mixed-effects model was used to analyze the variation trend of different dimensions of both SF-36 and FACT-BMT with different follow-up times.

**Results:**

Of the 425 participants, recipients of HID and MSD who survived more than 1 year (*n* = 230) were included in the final analysis of QoL (median age [range]: 36, [15, 66]). The 3 year overall survival (OS) of HID and MSD was 82.42% and 86.46%, respectively. QoL was assessed using both SF-36 and FACT-BMT and there was longitudinal recovery with clinical significance in the cohort. Compared to MSD-HSCT patients, HID-HSCT recipients demonstrated superior QoL performance in some subscales describing physical and mental wellness. Specifically, the difference in physical performance is more remarkable using FACT-BMT whereas that in mental wellness is more significant using SF36. In the subsequent stratified analysis, patients with a history of aGVHD or CMV reactivation demonstrated inferior QoL.

**Conclusions:**

Long-term survivors of HID HSCT achieved better QoL in some sub-scales compared to MSD HSCT. In addition, SF-36 and FACT-BMT demonstrated different performance thus combination of both improved capacity of the evaluation system.

**Supplementary Information:**

The online version contains supplementary material available at 10.1186/s12967-022-03803-y.

## Key points

- Long-term survivors of HSCT recipients demonstrated longitudinal recovery in QoL which can be quantified with both SF-36 and FACT-BMT forms
- Recipients of HID-HSCT reported higher performance of QoL in both mental and physical dimensions.
- History of acute GVHD or CMV reactivation significantly was associated with inferior QoL.
- The SF-36 and FACT-BMT forms demonstrated different performance in the quantification of QoL recovery and combination of both may improve QoL evaluation system

**Supplementary Information:**

The online version contains supplementary material available at 10.1186/s12967-022-03803-y.

## Background

Allogenic hematopoietic stem cell transplantation (allo-HSCT) is a curative option for patients with hematological malignancies and some non-malignant hematological diseases. A high quality of life (QoL) is crucial for the wellness of long-term survivors [1–3]. The QoL is well acknowledged as multidimensional parameters including physical, emotional, social performance and well-being from patients’ perceptions [2]. QoL assessment helps healthcare providers to evaluate clinical interventions and is also an integral component in estimating medical outcome [4].

Numbers of haploidentical donor HSCTs (HID-HSCTs) are increasing rapidly due to decreasing family size and have become the largest source of allo-HSCT donors in China [5]. Since haploidentical donors are immediately available to the majority of patients, HID-HSCT significantly extends treatment choice and demonstrates comparable clinical outcomes as compared to conventional HSCT [5–7]. However, QoL represents a major concern in long-term survivors considering high incidence of complications following HID-HSCT [8, 9]. A number of studies have evaluated QoL in HID-HSCT recipients with inconsistent findings [6, 10]. While some reports studied QoL in the setting of HID-HSCT, control groups were heterogeneous which included HLA-matched sibling, matched unrelated, and unrelated umbilical cord blood donors [11]. In addition, the majority of surveys were retrospective and QoL was not assessed with comprehensive questionnaires including HSCT-specific scales [12]. To our knowledge, large-scale prospective study is lacking that focused on longitudinal changes in QoL in HID-HSCT recipients using multiple HSCT-specific questionnaires.

Our center has established a system for the assessment of QoL in HSCT recipients with the advantages of high specificity, high sensitivity, high acceptance and easy follow-up [13]. In our previous study, we used the Mos 36-Item Short-Form Health Survey (SF-36) to describe the trajectory of QoL recovery and found significant improvement in QoL among one-year survivors (> 1 year after HSCT). In this prospective cohort study, we aim to establish a comprehensive QoL evaluation system for long-term survivors (> 1 year) making use of both SF36 and The Functional Assessment of Cancer Therapy Bone Marrow Transplant (FACT-BMT). General QoL was evaluated by SF36, a well-established QoL measurement that is often used in cancer populations [14]. HSCT-specific QoL was evaluated with the FACT-BMT scale, a self-administered tool used to assess multidimensional domains of QoL in HSCT recipients [15, 16].

The primary end point of this study was to investigate the differences in QoL trajectory between HID-HSCT and MSD-HSCT during long-term follow-up. The secondary end point was to investigate the feasibility and practicability of the QoL assessment system with a combination of SF36 and FACT-BMT.

## Methods

### Study design and participants

**Fig. 1:**
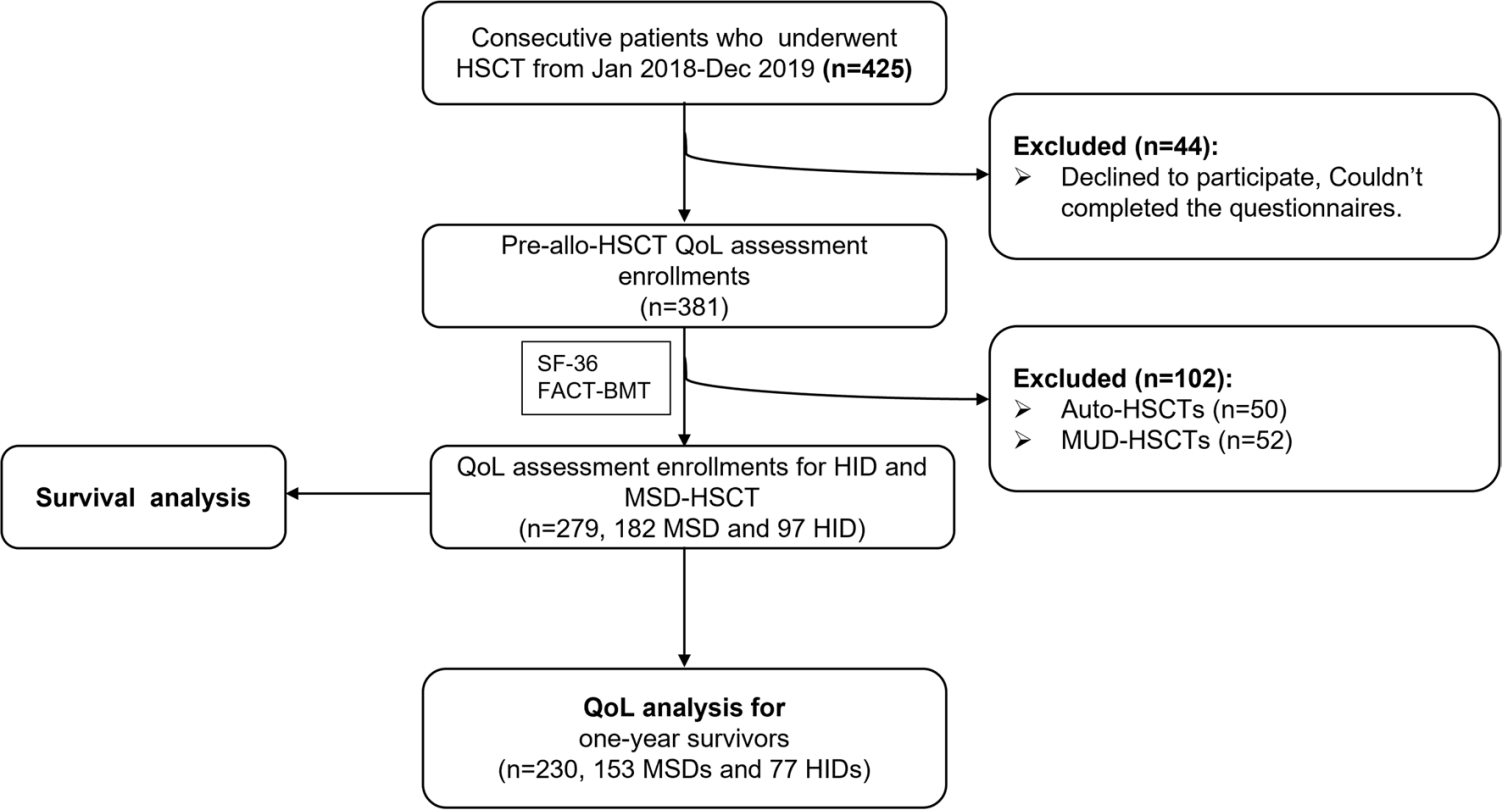
Flowchart of enrollment and analysis of participants

This prospective study enrolled consecutive patients (*n* = 425) who had received HSCT between January 2018 and December 2019 at the HSCT Center, Blood Disease Hospital, Chinese Academy of Medical Sciences and Peking Union Medical College (CAMS&PUMC). Participants were informed of the objective of the study and asked to complete QoL questionnaires including the SF-36 and the FACT-BMT (version 4). In the present study, 44 patients were excluded due to declination to participate or inability to complete questionnaires independently. A total of 25 patients dropped off during follow-up. The total response rate and follow-up rate reached 89.6% and 93.4% respectively. A number of 279 patients received MSD HSCT (*n* = 182) or HID HSCT (*n* = 97). Patients who survived 1 year after transplant (*n* = 230) were included for the analysis of QoL. A detailed study flow chart is shown in Fig. 1.

All participants signed written informed-consent forms and completed questionnaires online at their earliest convenience. All procedures were in accordance with the ethical standards of the institutional and national research committee (Ethics committee of Blood Disease Hospital, Chinese Academy of Medical Sciences + KT2013019001-EC-1) and with the 1964 Helsinki Declaration and its later amendments or comparable ethical standards. The institutional review board approved all study procedures and forms.

### Transplantation regimen

For acute myelogenous leukemia (AML) patients, the myeloablative conditioning regimen consists of fludarabine (30 mg/m^2^/d, days − 6 to − 4), busulfan (3.2 mg/kg/d, days − 9 to − 7), cyclophosphamide (40 mg/kg/d, days− 3 to − 2) and cytarabine (2 g/m^2^/d, days − 9 to − 7). For myelodysplastic syndrome/myeloproliferative neoplasm (MDS/MPN) patients, the 5-day decitabine-included regimen was used including decitabine (20 mg/m^2^/d, days − 9 to − 5), fludarabine (30 mg/m^2^/d, days − 6 to − 4), busulfan (3.2 mg/kg/d, days − 9 to − 7), cyclophosphamide (40 mg/kg/d, days − 3 to − 2) and cytarabine (2 g/m^2^/d, days  9 to − 7). For acute lymphocyte leukemia (ALL) patients, total body irradiation (TBI) based myeloablative conditioning was administered: TBI (3.3 Gy/d, days − 9 to − 7), cyclophosphamide (40 mg/kg/d, days − 6 to − 5), fludarabine (30 mg/m^2^/d, days − 4 to − 2) and cytarabine (2 g/m^2^/d, days − 4 to − 2). The conditioning regimen for aplastic anemia (AA) patients includes FAC and BFAC. The FAC conditioning regimen was composed of fludarabine (30 mg/m2/d, days − 5 to − 1), cyclophosphamide (30 mg/kg/d or 37.5 mg/kg/d, days − 5 to − 2) and rabbit antilymphocyte globulin (rATG) (Thymoglobulin^®^, Genzyme, Cambridge, MA) (2.5 mg/kg/d, days − 5 to − 1) or porcine antilymphocyte globulin (pALG) (Anti-lymphocyte Immunoglobulin®, Wuhan Institute of Biological Products Co.,Ltd., China) (25 mg/kg/d, days − 5 to − 1). The BFAC conditioning regimen included busulfan (3.2 mg/kg/d, days − 7 to − 6) on the basis of FAC regimen. All haplo-identical recipients received rATG (2.5 mg/kg/day, days − 5 to − 2) or pALG (20 mg/kg/day, days − 5 to − 2).

The GVHD prophylaxis was cyclosporin (CSA) or tacrolimus (FK506) (from day − 1) based [17–19]. Methotrexate was used for 3 doses (days + 1, + 3 and + 6) in MSD-HSCT and 4 doses (days + 1, + 3, + 6 and + 11) for HID HSCT. Mycophenolate mofetil was added for HID-HSCT recipients which initiated at 500 mg twice daily on day-9 and was titrated slowly downward until day + 60 and discontinued on day + 90. Details of conditioning regimen, GVHD prophylaxis and treatment strategy, supportive care were referred to our previously published reports [17–19].

### Data collection

Social-demographic variables were recorded including personal code, age, gender, clinical diagnoses, marital status, financial burden, insurance, employment status, and insurance payment. Clinical variables were recorded in the clinical records and analyzed upon completion of the study including primary disease, chromosome karyotype, conditioning regimen, date of transplant, HSCT type, stem cell source, minimal residual disease (MRD) before HSCT, Eastern Cooperative Oncology Group performance score (ECOG score), Hematopoietic Cell Transplant-Comorbidity Index (HCT-CI) score and infection The assessment of acute and chronic GVHD was conducted at real time by clinical teams. Acute graft versus host disease (aGVHD) and chronic graft versus host disease (cGVHD) were evaluated using international criteria [20, 21].

### QoL assessment

This prospective study was designed for routine examination of QoL for recipients of MSD and HID-HSCT. All participants were asked to complete a questionnaire before transplantation and at scheduled time points after transplantation including 3 months, 6 months, 1 year, 1.5 years, 2 years, 3 years, and 5 years. Of the remaining 230 one-year survivors, there were 230 (100%), 230 (100%), 229 (99.57%), 116 (50.43%), 165 (71.74%) responded to our questionnaire at different timepoints, including 3 months, 6 months, 1 year, 2 year and more than 2 years, respectively. All participants were asked to complete both SF-36 Form and FACT-BMT. SF36 From, a 36-item, generic questionnaire that assesses the functional status and well-being on eight multi-item subscales: physical functioning (PF), role physical (RP), bodily pain (BP), general health (GH), vitality (VT), social functioning (SF), role emotional (RE), and mental health (MH). The FACT-BMT used the subscales including physical well-being (PWB), functional well-being (FWB), social well-being (SWB), emotional well-being (EWB), BMT scale (BMTS), and total with BMT module (FACT-BMT). Patients completed the questionnaires on an online applet named *HSCT-QoL-CLOUD* which sends survey notifications to patients at scheduled timepoint. The results could be stored in the cloud and exported to the database.

### Statistical analysis

All statistical analyses were performed using Stata SE 16.0 (Stata Corp, College Station, Texas). A 2-sided *P* < 0.05 was considered to indicate statistical significance. Baseline characteristics of the study population were analyzed by chi-square test for categorical variables and t-test or Wilcoxon rank-sum tests for continuous variables.

Kaplan–Meier and Laplace regression models were used to estimate and compare the median survival time (months) and 95% confidence intervals (CIs) according to different HSCT types. Cox proportional hazards regression models were used to estimate the hazard ratios (HRs) and 95% CIs for the incidence of death according to HSCT type. Follow-up time (months) was calculated as the time from study entry to death or the final examination. The linear mixed-effects model estimating the β-coefficients and 95% confidence intervals (CIs) was used to analyze the variation trend of different dimensions of both SF-36 and FACT-BMT with different follow-up times. The fixed effect included HSCT, follow-up time (month), and their interaction. Stratified analyses were performed to explore the association between HSCT and QoL according to incident aGVHD and cytomegalovirus (CMV) reactivation during the follow-up. All models were adjusted for age, sex, education, body mass index (BMI), main caregivers, diagnosis, and transplantation type, history of aGVHD and, cGVHD, and history of infection.

## Results

### Basic characteristics of HSCT recipients

This study included 279 participants (median age: 36, ranging from 15 to 66, 49.1% female) who received HID (*n* = 97) or MSD (*n* = 182) HSCT at our center (Table 1). The majority of underlying diseases are hematological malignancies (93.1%) and all patients reached full chimerism post-HSCT. More than half of the patients (66.25%) were fully active before HSCT (ECOG score 0). Most patients (93.54%) were evaluated HCT-CI score ranging from 0 ~ 1 at HSCT. A total of 80 patients didn’t achieve hematologic response or retained untreated before HSCT. Most patients (96.42%) accepted school education for more than 6 years and all patients were able to complete the QoL questionnaires independently. While 72.04% of patients were married, spouses are main caregivers for 54.8% of patients. HID-HSCT recipients received more potent immunosuppressive regimens for the prophylaxis of GVHD. More patients in HID group were taken care of by their spouses (54.64%). No significant difference was documented between MSD and HID-HSCT groups in terms of HCT-CI score, ECOG score, and hematologic responses before HSCT. As we have mentioned in the method session, HID-HSCT recipients received more potent immunosuppressive regimens for the prophylaxis of GVHD, including the addition of ATG in the conditioning regimen, extension use of CSA/FK506/MTX and the addition of mycophenolate mofetil after transplant. The basic characteristics and its comparison between HID and MSD were shown in Table 1.

**Table 1:** Difference of clinical characteristics between patients underwent MSD-HSCT and haploidentical HSCT (HID-HSCT

Characteristics	All patients (n = 279)		*P*	One-year survivors (n = 230)		*P*
	MSD-HSCT (N = 182)	HID-HSCT (N = 97)		MSD-HSCT (N = 153)	HID-HSCT (N = 77)	
Age	37.87 (11.59)	33.07 (12.32)	0.00^b^	37.71 (11.438)	34.25 (11.703)	0.03^b^
Gender Male	90 (49.45)	51 (52.58)	0.71	77 (50.33)	38 (49.35)	1.00
BMI ≥ 25	58 (31.87)	29 (29.90)	0.79	46 (30.07)	25 (32.47)	0.76
Education (years)			0.42			0.67
< 6	5 (2.75)	6 (6.19)		5 (3.27)	4 (5.19)	
6 ~ 12	124 (68.13)	60 (61.86)		102 (66.67)	48 (62.34)	
> 12	53 (29.12)	32 (32.99)		46 (30.07)	25 (32.47)	
Marital status			0.02^b^			0.08
Single	39 (21.43)	35 (36.08)		31 (20.26)	26 (33.77)	
Married	141 (77.47)	60 (61.86)		120 (78.43)	50 (64.94)	
Divorced/widowed	2 (1.10)	2 (2.06)		2 (1.31)	1 (1.29)	
Main caregivers			0.02^b^			0.03^b^
Spouse	74 (40.66)	53 (54.64)		59 (38.56)	42 (54.55)	
Alternative	109 (59.89)	44 (45.36)		94 (61.44)	35 (45.45)	
Family income (rmb/year)			0.15			0.08
< 100000	81 (44.51)	33 (34.02)		69 (45.10)	24 (31.17)	
≥ 100000	94 (51.65)	57 (58.76)		79 (51.63)	48 (62.34)	
Source of medical payment			0.36			0.89
Insurance	111 (60.99)	64 (65.98)		96 (62.75)	49 (63.63)	
Self-payment	70 (38.46)	31 (31.96)		56 (36.60)	27 (35.06)	
Diagnosis			0.01^b^			0.09
AML/ALL	117 (64.29)	79 (81.44)		98 (64.05)	61 (79.22)	
MDS/MPN	48 (26.37)	16 (16.49)		42 (27.45)	14 (18.18)	
AA	15 (8.24)	2 (1.10)		11 (7.19)	2 (2.60)	
Lymphoma	2 (1.10)	0		2 (1.31)	0	
Cytogenetic Data			0.57			0.29
Normal	121 (66.48)	68 (70.10)		102 (66.67)	58 (75.32)	
Monosomal karyotype	32 (17.58)	11 (11.34)		29 (18.95)	7 (9.09)	
Complex karyotype	18 (9.89)	12 (12.37)		14 (9.15)	8 (10.39)	
Unable to define the karyotype	11 (6.04)	6 (6.19)		8 (5.23)	4 (5.19)	
Hematologic Response at HSCT			1.00			0.88
CR	130 (71.43)	69 (71.13)		110 (71.90)	54 (70.13)	
Non-CR or untreated	52 (28.57)	28 (28.87)		43 (28.10)	23 (29.87)	
ECOG Performance Score			0.89			0.31
0 (fully active)	121 (66.48)	66 (68.04)		95 (62.09)	54 (70.13)	
> 0	61 (33.52)	31 (31.96)		58 (37.91)	23 (29.87)	
HCT-CI			0.49			0.17
0 ~ 1	172 (94.50)	89 (91.75)		145 (94.77)	70 (90.91)	
2	10 (5.49)	8 (8.25)		8 (5.23)	7 (9.09)	
GVHD prophylaxis			0.18			0.16
CSA-based	156 (85.71)	77 (79.38)		135 (88.24)	62 (80.52)	
FK506-based	26 (14.29)	20 (20.62)		18 (11.76)	15 (19.48)	
Use of ATG	75(41.21)	97(100)	0.00^b^	63(41.18)	77(100)	0.00^b^
Blood type			0.80			0.78
Match	75 (41.21)	37 (38.14)		63 (41.18)	30 (38.96)	
Mismatch	108 (59.34)	59 (60.82)		90 (58.82)	47 (61.04)	
Neutrophil engraftment	14.62 (15.10)	18.15 (24.17)	0.31	14.96 (16.422)	15.11 (3.272)	0.94
Platelet engraftment	16.34 (13.19)	17.86 (8.45)		16.8 (14.295)	19 (8.785)	0.22
Infection						
Bacteria	69 (37.91)	34 (35.05)	0.70	74 (48.37)	28 (36.36)	0.08
Fungus	34 (18.68)	30 (30.93)	0.02^b^	18 (11.76)	7 (10.87)	0.54
CMV	55 (30.22)	41 (42.27)	0.04^b^	33 (21.57)	26 (33.77)	0.04^b^
aGVHD	64 (35.16)	53 (54.64)	0.00^b^	52 (33.99)	44 (57.14)	0.00^b^
cGVHD	26 (14.29)	11 (11.34)	0.58	26 (16.99)	10 (12.99)	0.43

*BMI* body mass index, *AML* acute myelogenous leukemia, *ALL* acute lymphocyte leukemia, *MDS/MPN* myelodysplastic syndrome/Myeloproliferative Neoplasm, *AA* aplastic anemia, *CR* complete remission, *ECOG* eastern cooperative oncology Group, *HCT-CI* hematopoietic cell transplant-comorbidity Index (HCT-CI) score, *HSCT* hematopoietic stem cell transplantation, *MSD* matched sibling donor, *HID* haplo-identical donor, *PB* peripheral blood, *BM* bone marrow, *CMV* cytomegalovirus, *aGVHD* acute Graft versus Host Disease, *cGVHD*: chronic graft versus host disease, *CSA* cyclosporine, *FK506* tacrolimus

^a^Values were expressed as n (%) or median (interquartile range)

^b^Indicates the statistical significance for the factors

### HID-HSCT showed similar survival with higher incidence of aGVHD and CMV reactivation

**Fig. 2:**
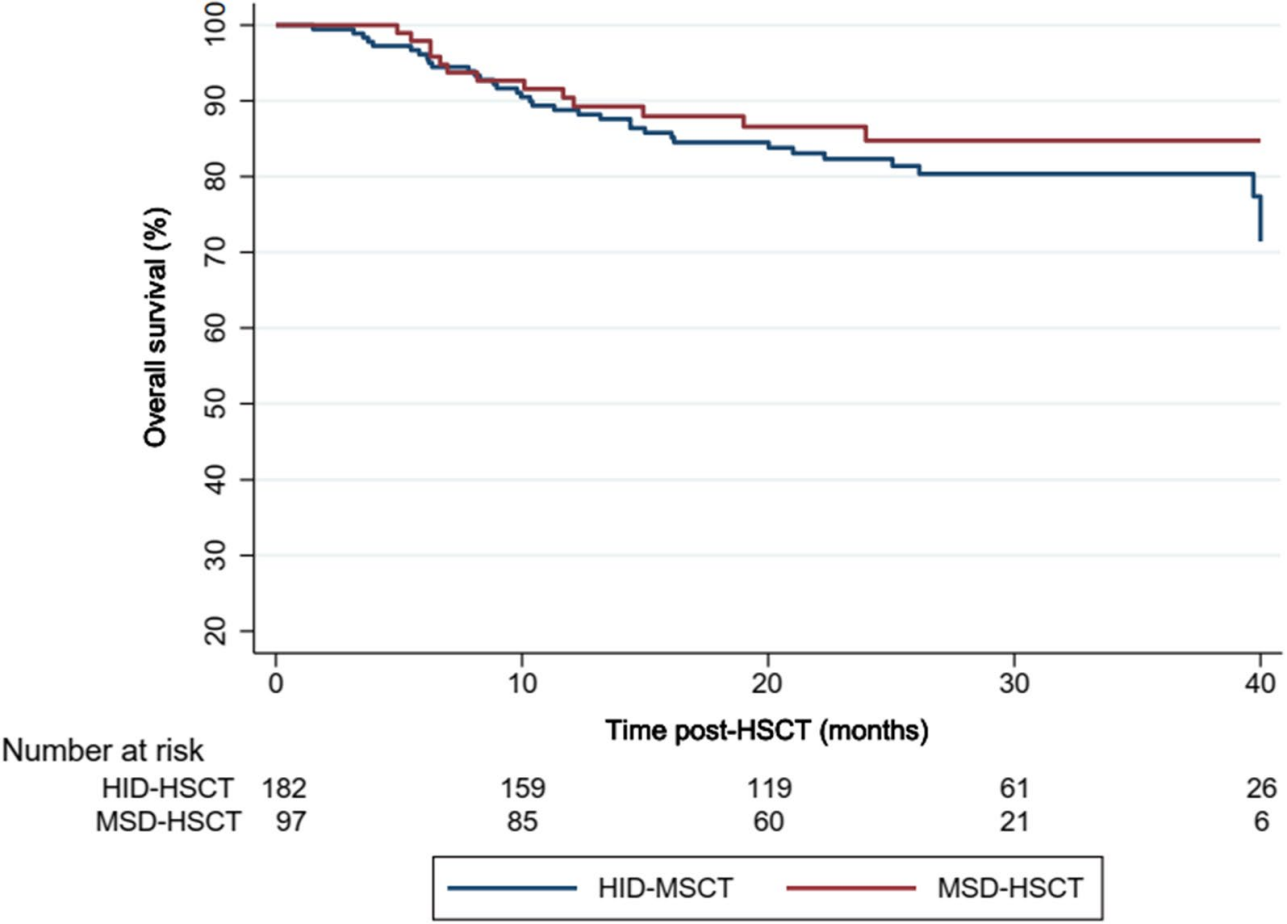
Survival analysis of MSD- and HID-HSCT recipients

We firstly compared the overall outcome of the HID-HSCT and MSD-HSCT recipients. After a median follow-up of 24.33 months (95% CI 23.6–25.1 months), overall survival (OS) was comparable which was 82.42% and 86.46% at 3 years in all recipients of HID and MSD HSCT respectively (Fig. 2). Consistently, mortality rate in HID-HSCT was comparable to MSD-HSCT with HR (95% CI) at 0.77 (0.37–1.61) in a multi-adjusted COX model. Despite the similarity in survival, significantly higher proportion of HID-HSCT recipients had a history of acute GVHD (57.1% vs 34.0%, *p* = 0.001) and CMV reactivation (33.8% vs 21.6%, *p* = 0.045) which may affect the QoL (Table 1).

Additional factors that may contribute to survival or QoL were subsequently analyzed. Unsurprisingly, HID-HSCT recipients were significantly younger than recipients of MSD-HSCT however the difference was very small (34.25 [11.703] vs 37.71 [11.438], mean [SD], p = 0.032), which we didn’t explore further. Of note, more than half of the HID patients were taken care of by their spouses (54.55%) whereas more patients in the MSD group were taken care of by alternative relatives. Other investigated factors were comparable between these two groups including gender, family income, body weight index (BMI), underlying diseases, performance status, school years, marital status, insurance sources, history of bacterial/fungal infections and history of chronic GVHD (Table 1).

### QoL recovered longitudinally using both SF-36 and FACT-BMT

**Fig. 3:**
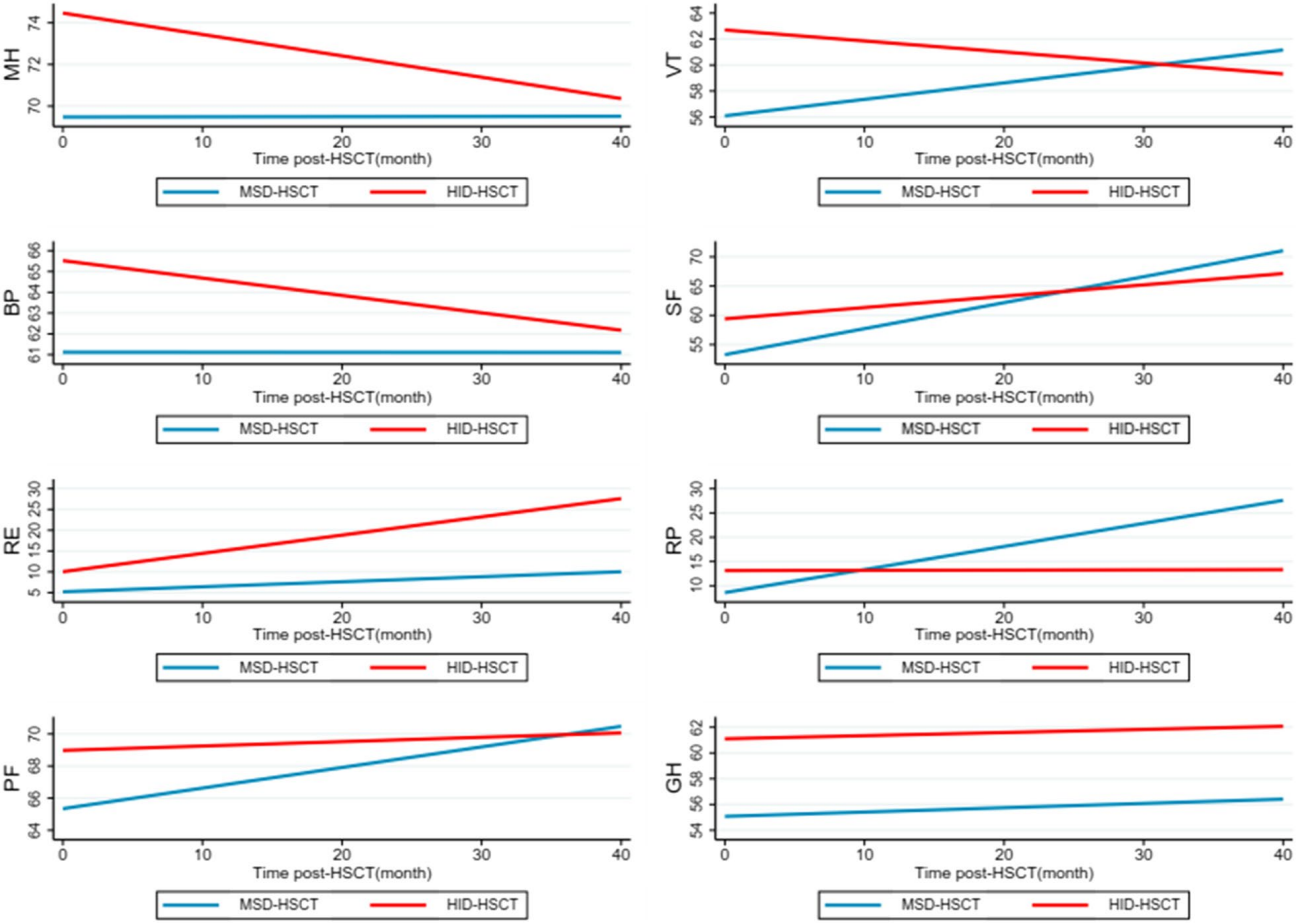
QoL trajectories in SF-36 dimensions between MSD- and HID-HSCT groups. Trajectories represent β-coefficients from linear mixed-effect models adjusted for age, sex, education, body mass index (BMI), main caregivers, diagnosis, transplantation type, history of aGVHD and cGVHD, and history of infection. MSD-HSCT group as reference group.

**Fig. 4:**
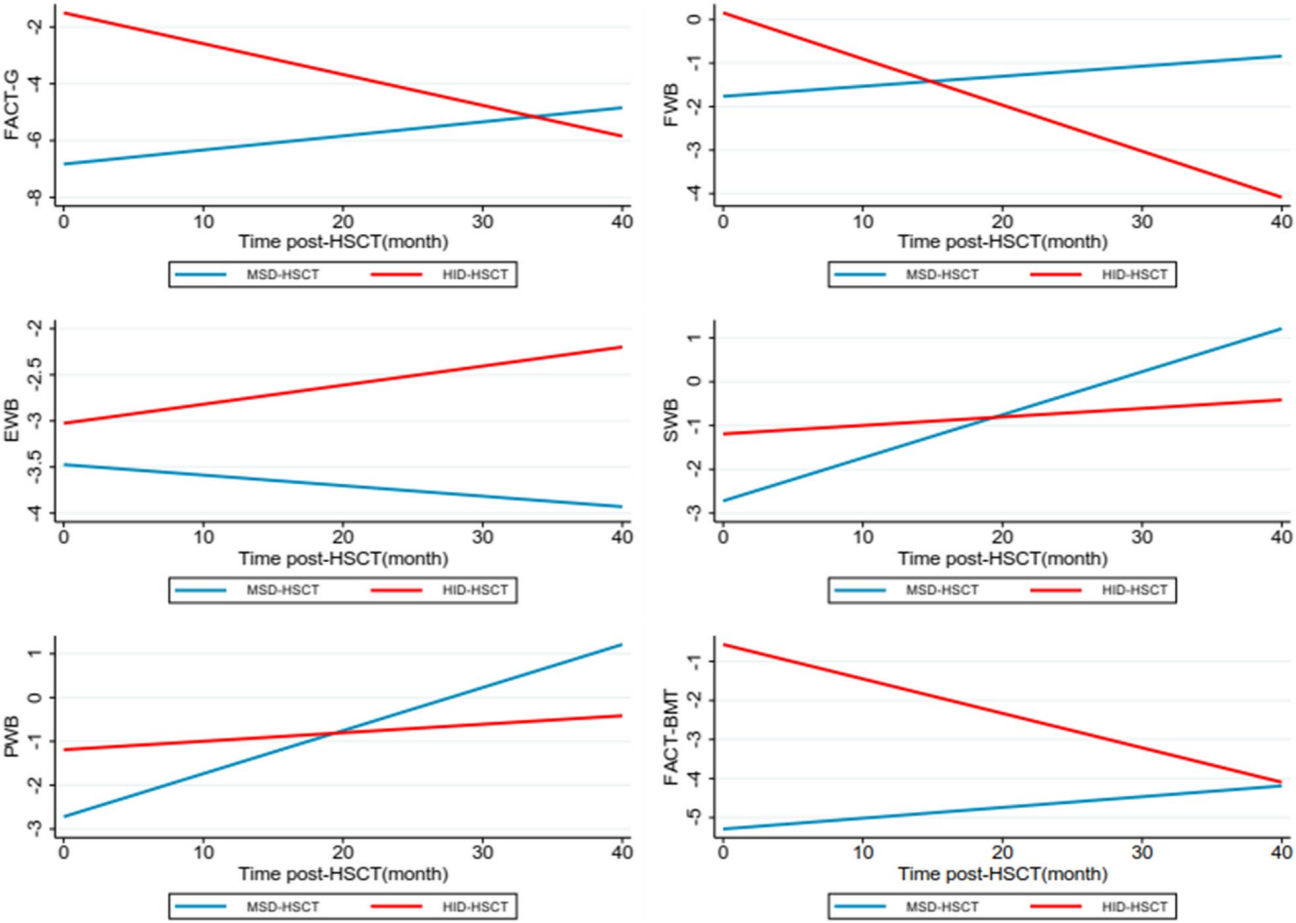
QoL trajectories in FACT-BMT dimensions between MSD- and HID-HSCT groups. Notes: Trajectories represent β-coefficients from linear mixed-effect models adjusted for age, sex, education, body mass index (BMI), main caregivers, diagnosis, transplantation type, history of aGVHD and cGVHD, and history of infection. MSD-HSCT group as reference group

We then investigated the longitudinal recovery of QoL in the whole cohort during the follow-up. We have described the QoL values for the HID-HSCT and MSD-HSCT groups at each time point. According to the results, scores of most dimensions increased gradually after transplantation. For instance, the mean score of RP has been raised from 12.82 to 23.33 in the MSD-HSCT group, and from 16.25 to 20.00 in the HID-HSCT group (Additional file 1: Table S1). We performed the linear mixed-effects model to analyze the variation trend of different dimensions with different follow-up times. Using the SF-36 form, scores on most scales increased gradually over time (Fig. 3, Table 2), including physical functioning (PF), role physical (RP), bodily pain (BP), general health (GH), vitality (VT), social functioning (SF), and role emotional (RE). Of note, statistically significant difference was observed in RP (*P* < 0.001), SF (*P* < 0.001) and PF (*P* = 0.011) indicating significant recovery mainly on physical function. However, we didn’t observe significant improvement in mental health (MH) score over time (β = − 0.07, 95% CI: − 0.2 to 0.08). Consistently, most of the scales of FACT-BMT form also improved over time (Fig. 4, Table 2). Similarly, physical well-being (PWB), the dimensions evaluating recovery of physical function, also reached clinically relevant significance. Thus, both forms demonstrated longitudinal improvement of QoL post-HSCT, especially in physical function related scales.

**Table 2:** Differences in longitudinal quality of life (QoL) assessed by SF36 and FACT-BMT Form between HID and MSD patients

QoL	HSCT (HID vs. MSD)		Time (months post-HSCT)	
	β (95% CI) ^a^	*P*	β (95% CI) ^a^	*P*
SF-36				
GH	5.46 (0.62, 10.29)	0.027^b^	0.03 (− 0.15, 0.22)	0.722
PF	2.83 (− 3.15, 8.82)	0.353	0.30 (0.07, 0.52)	0.011^b^
RP	4.06 (− 1.83, 9.94)	0.177	0.82 (0.44, 1.20)	< 0.001^b^
RE	4.83 (− 4.41, 14.08)	0.306	0.33 (-0.10, 0.76)	0.129
SF	4.21 (− 2.08, 10.51)	0.190	0.68 (0.41, 0.94)	< 0.001^b^
BP	4.06 (− 0.79, 8.91)	0.101	0.09 (− 0.09, 0.27)	0.336
VT	6.18 (1.37, 11.00)	0.012^b^	0.12 (− 0.05, 0.29)	0.168
MH	4.53 (0.38, 8.68)	0.032^b^	− 0.07 (− 0.20, 0.08)	0.333
FACT-BMT				
PWB	1.49 (0.11, 2.87)	0.034^b^	0.09 (0.04, 0.14)	< 0.001^b^
SWB	1.29 (0.21, 2.38)	0.020^b^	− 0.05 (− 0.10, 0.01)	0.099
EWB	0.47 (− 0.57, 1.50)	0.378	− 0.01 (− 0.04, 0.02)	0.482
FWB	1.89 (0.52, 3.26)	0.007^b^	0.02 (− 0.06, 0.10)	0.560
FACT-G	5.34 (1.72, 8.76)	0.004^b^	0.05 (− 0.11, 0.20)	0.545
TOI	2.80 (0.43, 5.18)	0.021^b^	0.09 (− 0.01, 0.19)	0.095
FACT-BMT	4.64 (1.09, 8.19)	0.010^b^	0.03 (− 0.12, 0.17)	0.727

*GH* general health, *PF* physical functioning, *RP* role physical, *RE* role emotional, *SF* social functioning, *BP* bodily pain, *VT* vitality, *MH* mental health, *PWB* physical well-being, *FWB* functional well-being, *SWB* social well-being, *EWB* emotional well-being, *FACT-BMT* total with BMT module, TOI, FACT-BMT Trial Outcome Index (TOI)

^a^Model adjusted for age, sex, education, body mass index (BMI), main caregivers, diagnosis, transplantation type, history of aGVHD and cGVHD, and history of infection

^b^Indicates the statistical significance for the factors

### Recipients of HID reported superior QoL

We next ask if there was difference between recipients of HID- and MSD-HSCT. These two cohorts reported similar scores of QoL before transplantation (Table 2). Compared to MSD-HSCT recipients, HID-HSCT recipients exhibited better performance in physical dimensions using FACT-BMT form including global FACT score (FACT-G), physical well-being (PWB), social well-being (SWB), functional well-being (FWB) and FACT-BMT Trial Outcome Index (TOI). Using SF-36 form, the HID-HSCT recipients demonstrated superior advantage in GH, and mental dimensions including VT and MH (Table 1). In sum, HID-HSCT recipients demonstrated higher performance of QoL after HSCT in terms of physical and mental scales and the difference was more remarkable using FACT-BMT Form.

### History of aGVHD and CMV reactivation compromised QoL recovery and attenuates the advantage of QoL in HID-HSCT

As more HID-HSCT recipients had a history of acute GVHD/CMV reactivation, we conducted stratified analysis to evaluate the effect of these complications on QoL recovery. In patients without a history of aGVHD, HID-HSCT recipients showed significantly greater recovery in both physical and mental scales especially for GH, SF, VT and MH items (Additional file 1: Table S2). For patients with a history of aGVHD, HID-HSCT maintained the advantages albeit at smaller difference in VT and physical dimensions (GH and BP) while demonstrated inferior scores for SF without statistical significance (β = − 3.19, 95% CI − 13.36 to 6.98). When FACT-BMT Form was used, HID-HSCT recipients with a history of aGVHD also showed significantly higher FWB subscale score (β = 2.51, 95% CI: 0.19 to 4.85) whereas lost the superiority for the rest of domains (PWB, SWB, FWB, FACT-G, TOI and FACT-BMT).

For patients without a history of CMV reactivation, recipients of HID-HSCT had significantly better scores on the PWB, SWB, FWB, FACT-G, TOI, and FACT-BMT scales using FACT-BMT. However, all these advantages were lost when there was a history of CMV reactivation. When SF36 Form was used, HID-HSCT recipients without CMV reactivation history demonstrated a trend toward worse QoL recovery including PF, RE, SF and VT in the context of CMV reactivation which further confirmed the detrimental effect of CMV infection on QoL (Additional file 1: Table S3). In sum, HID-HSCT recipients demonstrated superior QoL performance, but the advantages were compromised by the incidence of aGVHD or CMV reactivation.

### Combination of SF-36 and FACT-BMT is superior for the evaluation of QoL

As we have shown, either SF-36 or FACT-BMT is competent to describe the longitudinal QoL recovery in long-term survivors of HSCT. However, for the comparison of QoL between HID and MSD-HSCT cohorts, these two evaluation systems exhibited diverse point of focus. SF36 depicted the advantage of HID-HSCT in GH and mental subscales including VT and MH. Otherwise, FACT-BMT showed the higher performance in physical dimensions including global FACT score (FACT-G), physical well-being (PWB), social well-being (SWB), functional well-being (FWB) and FACT-BMT Trial Outcome Index (TOI) in HID-HSCT recipients. The difference for GH and mental domain scores were greater using SF36 whereas superiority in the recovery for functional/physical domains was more profound when FACT-BMT was used. Furthermore, both forms are able to show the effect of aGVHD and CMV reactivation on QoL recovery despite difference in performance.

## Discussion and conclusions

In the present study, we combined SF36 and FACT-BMT to establish a comprehensive QoL assessment system and found that physical/functional scales (spanning SF-36 and FACT-BMT) significantly improved over time in a cohort of Chinese patients. Surprisingly, HID-HSCT cohort demonstrated better performance in QoL. Comprehensive assessment of QoL thus contributes to the improvement of health care and benefit recipients of HSCT.

QoL is a major concern for long-term survivors of HSCT which significantly affect their wellbeing. We have previously showed significant recovery of QoL one-year after HSCT using the SF36 form [13]. In this study, we aim to optimize treatment-specific tools in our QoL evaluation system by using a comprehensive scale (SF36) and a disease-specific scale (FACT-BMT), which has been adopted for quantifying patient-reported outcomes [22, 23]. Combination of the two forms enhances the ability to detect patients' perception of health status and increase comparability in patients specifically associated with HSCT [23]. In our study, the two questionnaires demonstrated good correlations in most domains in describing the trend of QoL recovery. Of note, SF36 and FACT-BMT exhibited differential performance in detecting differences in physical and mental dimensions respectively between the two study cohorts. Furthermore, the high response rate and low drop-off rate in the present study confirmed the feasibility to combine SF36 and FACT-BMT for the evaluation of QoL. The high compliance is also attributable to the application of applet which is superior to traditional hard mails by enabling timely notifications and immediate accessibility [22]. Hence, combination of these two forms represent a feasible and powerful approach for the evaluation of QoL in recipients of HSCT.

Haploidentical donors represent the largest source of allo-HSCTs in China since the last decade. HID-HSCT has clinical outcomes similar to that of MSD- or MUD-HSCT for patients with AML, ALL, MDS, and SAA [5, 6, 24, 25]. HID-HSCT is especially beneficial for patient with high-risk leukemia or elderly patients with young offspring donors, attributable to an association with lower incidence of relapse [7]. However, its apparently higher incidence of complications [26] such as GVHD warrants deeper investigation of the longitudinal recovery of QoL in this setting. Only a few longitudinal studies assessed the QoL recovery between recipients of HID-HSCT and MSD-HSCT [27, 28]. Nevertheless, most studies were performed retrospectively with high heterogeneity in the control groups [10, 11]. In this prospective study, we confirm that HID-HSCT has similar or superior recovery of QoL in long-term survivors as compared to the conventional MSD-HSCT. Notably, HID-HSCT patients reported favorable recovery of general health and emotional wellbeing. This is similar to the “post-traumatic growth” theory [29, 30] for example, recipients of allo-HSCT demonstrated better mental status compared to recipients of chemotherapy. In our study, patients receiving HID-HSCT lack matched sibling donors, or experienced more or severer post-HSCT complications, which represents a traumatic stressor [31]. This may partially contribute to the superior QoL recovery in recipients of HID-HSCT.

We furtherly performed stratified analysis to analyze the effect of post-HSCT complications on QoL recovery, as higher incidences of complications such as aGVHD and CMV reactivation in HID-HSCTs with clinical significance. History of GVHD represents a risk factor of inferior QoL post-HSCT, especially in physical and functional dimensions [9, 32, 33] [34] which is consistent with our finding. Our results also indicated an inverse association between aGVHD and the recovery of mental health. CMV reactivation remains a common complication despite advances in preemptive interventions and poses significant risk of morbidity and mortality [35]. Subsequent CMV infections incur longer hospitalization and profound economic burden [36, 37]. To our knowledge, we are the first to demonstrate the detrimental role of CMV reactivation on QoL recovery post-HSCT. In addition to the impairment of longitudinal QoL recovery, the advantages of HID-HSCT on QoL also lost in the context of aGVHD or CMV reactivation. Thus, these complications exert long-term effect on HSCT recipients in addition to the adverse effect on survival.

The limitation of this study is also clear including the nature of single-center study and the relatively limited enrollment. Despite this, the present study being conducted at the largest blood disease hospital in China, the participants demonstrated big variety and are representative. Larger scale, multicenter studies are thus warranted.

In sum, our study provides clear evidence that HID-HSCT can yield a considerate survival rate with ideal quality of life in long-term survivors thus extending the application of this transplant approach. Additionally, SF-36 and FACT-BMT have different performance in the quantification of QoL and combination of both improve the capacity of the evaluation system for QoL after HSCT.

## Supplementary Information

**Additional file 1: Table S1.** Longitudinal of QoL scores using SF-36 and FACT-BMT at each time post-HSCT. **Table S2.** Stratified analysis of QoL between HID and MSD patients in GVHD and no-GVHD groups.**Table S3.**Stratified analysis of QoL between HID and MSD patients in CMV and no-CMV reactivation groups.

## Data Availability

All data generated or analyzed during this study are included in this published article.

## Abbreviations

Allo-HSCT: Allogenic hematopoietic stem cell transplantation
QoL: Quality of life
HID-HSCT: Haploidentical donor HSCT
SF-36: Mos 36-Item short-form health survey
FACT-BMT: The functional assessment of cancer therapy bone marrow transplant
MSD-HSCT: Matched sibling donor HSCT
MRD: Minimal residual disease
aGVHD: Acute graft versus host disease
cGVHD: Chronic graft versus host disease
PF: Physical functioning
RP: Role physical
BP: Bodily pain
GH: General health
VT: Vitality
SF: Social functioning
RE: Role emotional
MH: Mental health
PWB: Physical well-being
FWB: Functional well-being
SWB: Social well-being
EWB: Emotional well-being
BMTS: BMT scale
FACT-BMT: Total with BMT module
CMV: Cytomegalovirus
BMI: Body mass index
ECOG score: Eastern cooperative oncology group performance score
HCT-CI: Hematopoietic cell transplant-comorbidity Index (HCT-CI) score
OS: Overall survival
AML: Acute myelogenous leukemia
ALL: Acute lymphocyte leukemia
MDS/MPN: Myelodysplastic syndrome/myeloproliferative neoplasm
AA: Aplastic anemia
PB: Peripheral Blood
BM: Bone marrow
TBI: Total body irradiation
FAC: Conditioning regimen consists of fludarabine, cyclophosphamide and ATG.
rATG: Rabbit antilymphocyte globulin
pALG: Porcine antilymphocyte globulin
CSA: Cyclosporin
FK506: Tacrolimus
MTX: Methotrexate
CR: Complete remission
