# Supplementary material for: Long-term survivors demonstrate superior quality of life after haploidentical stem cell transplantation to matched sibling donor transplantation
Source: J Transl Med. 2022 Dec 14;20:596. doi: 10.1186/s12967-022-03803-y (PMC9749359; doi:10.1186/s12967-022-03803-y)
Supplement: Supplementary file 1 — Additional file 1: Table S1. Longitudinal of QoL scores using SF-36 and FACT-BMT at each time post-HSCT. Table S2. Stratified analysis of QoL between HID and MSD patients in GVHD and no-GVHD groups.Table S3.Stratified analysis of QoL between HID and MSD patients in CMV and no-CMV reactivation groups. [file 12967_2022_3803_MOESM1_ESM.docx]

**Additional file 1: TableS1.** Longitudinal of QoL scores using SF-36 and FACT-BMT at each time post-HSCT.

| **HSCT** | **Time (m)** | **SF-36 (mean, 95%CI)** | | | | | | | |
| --- | --- | --- | --- | --- | --- | --- | --- | --- | --- |
|  |  | **GH** | **PF** | **RP** | **RE** | **SF** | **BP** | **VT** | [**MH**](mailto:c._95_zz@time_xg) |
| MSD-HSCT | 0 | 55.05 (52.49, 57.61) | 58.67 (55.21, 62.12) | 12.82 (9.17, 16.46) | 34.47 (28.59, 40.34) | 40.45 (36.93, 43.97) | 60.83 (57.55, 64.11) | 59.90 (57.30, 62.51) | 70.83 (68.70, 72.97) |
|  | 3 | 54.00 (49.11, 58.89) | 51.55 (45.12, 57.97) | 10.55 (3.80, 17.29) | 26.67 (16.28, 37.05) | 42.02 (35.8, 48.24) | 67.16 (62.84, 71.49) | 59.45 (54.34, 64.57) | 72.51 (68.19, 76.83) |
|  | 6 | 60.28 (54.07, 66.48) | 63.19 (55.64, 70.75) | 13.89 (5.35, 22.43) | 29.63 (16.39, 42.86) | 48.77 (40.51, 57.02) | 64.31 (57.01, 71.60) | 62.08 (55.34, 68.83) | 73.00 (67.29, 78.71) |
|  | 12 | 55.68 (47.28, 64.08) | 58.64 (46.54, 70.74) | 18.18 (3.82, 32.54) | 37.88 (19.98, 55.78) | 53.03 (42.01, 64.05) | 64.41 (56.39, 72.43) | 60.91 (52.91, 68.91) | 72.91 (65.44, 80.38) |
|  | 24 | 53.33 (49.60, 57.06) | 62.02 (56.52, 67.52) | 23.33 (15.22, 31.44) | 30.56 (21.61, 39.50) | 52.12 (46.23, 58.00) | 59.38 (54.63, 64.13) | 60.18 (56.53, 63.83) | 67.90 (64.68, 71.13) |
| HID  -HSCT | 0 | 58.88 (55.21, 62.56) | 58.88 (53.64, 64.13) | 16.25 (10.97, 21.53) | 38.39 (30.73, 46.05) | 42.16 (37.06, 47.26) | 65.23 (61.20, 69.26) | 63.57 (59.98, 67.16) | 74.71 (71.65, 77.77) |
|  | 3 | 60.34 (52.61, 68.08) | 49.83 (39.13, 60.53) | 9.66  (1.25, 18.06) | 32.18 (16.12, 48.25) | 42.53 (33.34, 51.72) | 65.38 (57.03, 73.73) | 62.76 (54.54, 70.98) | 75.45 (69.14, 81.75) |
|  | 6 | 61.04 (52.92, 69.16) | 53.75 (41.71, 65.79) | 10.00 (0.85, 19.15) | 16.67 (3.02, 30.31) | 45.37 (35.29, 55.45) | 58.67 (49.84, 67.49) | 59.17 (51.25, 67.09) | 73.67 (66.14, 81.20) |
|  | 12 | 59.00 (48.07, 69.93) | 71.50 (60.26, 82.74) | 22.00 (3.81, 47.81) | 26.67 (0.57, 53.91) | 58.89 (45.46, 72.31) | 65.60 (55.18, 76.02) | 60.00 (49.65, 70.35) | 64.80 (54.88, 74.72) |
|  | 24 | 65.31 (58.13, 72.50) | 52.97 (42.47, 63.47) | 20.00 (7.29, 32.71) | 59.37 (43.36, 75.39) | 46.53 (37.53, 55.53) | 68.00 (62.01, 73.99) | 61.41 (52.09, 70.73) | 77.00 (70.41, 83.59) |
|  |  |  |  |  |  |  |  |  |  |
| **HSCT** | **Time (m)** | **FACT-BMT (mean, 95%CI)** | | | | | | | |
|  |  | **PWB** | **SWB** | **EWB** | **FWB** | **FACT-G** | **BMT** | **TOI** | **FACT-BMT** |
| MSD-HSCT | 0 | 19.26 (18.41, 20.11) | 19.07 (18.24, 19.90) | 16.82 (16.25, 17.39) | 14.47 (13.52, 15.42) | 69.62 (67.33, 71.91) | 19.16 (18.68, 19.64) | 52.89 (51.39, 54.39) | 88.78 (86.49, 91.07) |
|  | 3 | 18.67 (16.95, 20.38) | 18.06 (16.35, 19.76) | 17.53 (16.29, 18.76) | 11.92 (10.32, 13.51) | 66.17 (61.96, 70.37) | 19.36 (18.33, 20.40) | 49.94 (47.42, 52.47) | 85.53 (81.49, 89.57) |
|  | 6 | 17.41 (14.70, 20.11) | 18.50 (16.43, 20.57) | 16.95 (15.39, 18.52) | 12.32 (9.52, 15.12) | 65.18 (58.26, 72.10) | 20.14 (18.92, 21.35) | 49.86 (45.18, 54.55) | 85.32 (78.66, 91.98) |
|  | 12 | 18.17 (14.93, 21.41) | 18.83 (16.23, 21.43) | 16.92 (14.71, 19.12) | 16.00 (13.01, 18.99) | 69.92 (60.83, 79.01) | 19.50 (17.27, 21.73) | 53.67 (47.85, 59.49) | 89.42 (79.99, 98.85) |
|  | 24 | 20.74 (19.09, 22.38) | 19.14 (17.82, 20.46) | 16.86 (15.80, 17.91) | 16.00 (14.17, 17.83) | 72.74 (67.96, 77.52) | 17.74 (16.69, 18.79) | 54.48 (51.49, 57.46) | 90.48 (85.78, 95.17) |
| HID-HSCT | 0 | 20.58 (19.47, 21.69) | 19.81 (18.85, 20.77) | 17.17 (16.08, 18.27) | 15.33 (13.97, 16.69) | 72.9 (69.68, 76.12) | 18.65 (17.95, 19.36) | 54.57 (52.61, 56.52) | 91.55 (88.47, 94.63) |
|  | 3 | 18.00 (15.37, 20.63) | 19.20 (17.23, 21.17) | 17.45 (15.54, 19.36) | 14.35 (11.39, 17.31) | 69.00 (61.16, 76.84) | 17.75 (16.05, 19.45) | 50.10 (45.90, 54.30) | 86.75 (79.89, 93.61) |
|  | 6 | 17.00 (14.17, 19.83) | 17.38 (13.88, 20.87) | 17.38 (13.96, 20.79) | 15.75 (11.71, 19.79) | 67.50 (57.53, 77.47) | 17.88 (16.12, 19.63) | 50.63 (46.32, 54.93) | 85.38 (76.48, 94.27) |
|  | 12 | 18.44 (13.91, 22.98) | 17.56 (15.67, 19.44) | 16.33 (13.43, 19.24) | 15.33 (10.29, 20.38) | 67.67 (55.42, 79.92) | 19.56 (17.73, 21.38) | 53.33 (44.99, 61.68) | 87.22 (75.37, 99.07) |
|  | 24 | 25.17 (21.71, 28.62) | 20.83 (17.38, 24.29) | 17.67 (14.73, 20.61) | 18.83 (11.19, 26.48) | 82.50 (70.21, 94.79) | 17.50 (16.28, 18.72) | 61.50 (52.65, 70.35) | 100.00 (88.5, 111.50) |

Abbreviations: GH, general health; PF, physical functioning; RP, role physical; RE, role emotional; SF, social functioning; BP, bodily pain; VT, vitality; MH, mental health; PWB, physical well-being; FWB, functional well-being; SWB, social well-being; EWB, emotional well-being, FACT-BMT, total with BMT module; TOI, FACT-BMT Trial Outcome Index (TOI).

**Additional file 1: TableS2.** Stratified analysis of QoL between HID and MSD patients in GVHD and no-GVHD groups.

| **QoL** | **No aGVHD** | | | | **aGVHD** | | | |
| --- | --- | --- | --- | --- | --- | --- | --- | --- |
|  | **HSCT (HID vs. MSD)** | | **Time (Months post-HSCT)** | | **HSCT (HID vs. MSD)** | | **Time(Months post-HSCT)** |  |
|  | **β (95% CI) ^a^** | ***P*** | **β (95% CI) ^a^** | ***P*** | **β (95% CI) ^a^** | ***P*** | **β (95% CI) ^a^** | ***P*** |
| **SF-36** | | | | | | | | |
| GH | 6.90 (0.08, 13.72) | 0.047^b^ | 0.06 (-0.16, 0.27) | 0.608 | 8.88 (1.06,16.70) | 0.026^b^ | 0.01 (-0.36, 0.38) | 0.957 |
| PF | 1.67 (-6.36, 9.71) | 0.684 | 0.28 (0.02, 0.54) | 0.033^b^ | 4.00 (-7.33, 15.32) | 0.489 | 0.35 (-0.10, 0.81) | 0.127 |
| RP | 5.54 (-3.76, 14.84) | 0.243 | 1.06 (0.57, 1.55) | 0.000^b^ | 2.60(-7.14, 12.35) | 0.601 | 0.40(-0.28, 1.08) | 0.249 |
| RE | 7.91(-4.99, 20.83) | 0.23 | 0.47(-0.09, 1.03) | 0.10 | 13.90 (-0.57, 28.39) | 0.06 | 0.31 (-0.48, 1.09) | 0.449 |
| SF | 11.99 (2.90, 0.52) | 0.010^b^ | 0.77 (0.52, 1.03) | 0.000^b^ | -3.19 (-13.36, 6.98) | 0.538 | 0.35 (-0.14, 0.85) | 0.162 |
| BP | 5.72 (-1.21, 12.64) | 0.106 | 0.11(-0.10, 0.33) | 0.308 | 7.74 (0.22, 15.26) | 0.044^b^ | 0.12 (-0.21, 0.47) | 0.480 |
| VT | 8.75 (1.67, 15.83) | 0.015^b^ | 0.15(-0.03, 0.34) | 0.104 | 7.66 (0.03, 15.29) | 0.049^b^ | 0.09 (-0.22, 0.42) | 0.565 |
| MH | 7.78 (2.08, 13.47) | 0.007^b^ | -0.09 (-0.24, 0.06) | 0.217 | 5.47 (-1.51, 12.45) | 0.124 | 0.10 (-0.17, 0.37) | 0.456 |
| **FACT-BMT** | | | | | | | | |
| PWB | 2.35 (0.40, 4.30) | 0.018^b^ | 0.07 (0.01,0.14) | 0.023^b^ | 1.67(-0.68, 4.01) | 0.164 | 0.12 (0.05, 0.19) | 0.001^b^ |
| SWB | 1.46 (-0.17, 3.09) | 0.000^b^ | 0.001 (-0.06, .06) | 0.994 | 0.27(-1.37, 1.93) | 0.742 | -0.15 (-0.25, -.04) | 0.007^b^ |
| EWB | 0.85 (-0.62, 2.32) | 0.257 | -0.02 (-0.06, 0.01) | 0.220 | 0.58(-1.13, 2.30) | 0.506 | 0.01 (-0.04, 0.06) | 0.716 |
| FWB | 2.33 (0.49, 4.17) | 0.013^b^ | 0.03 (-0.07, 0.14) | 0.841 | 2.51 (0.19, 4.85) | 0.034^b^ | 0.01 (-0.10, 0.12) | 0.84 |
| FACT-G | 7.49 (2.52, 12.47) | 0.003^b^ | 0.07(-0.14, 0.28) | 0.507 | 4.34 (-1.59, 10.29) | 0.151 | -0.01 (-0.24, 0.21) | 0.887 |
| TOI | 3.93(0.78, 7.08) | 0.015^b^ | 0.03 (-0.09, 0.17) | 0.554 | 2.79(-1.40, 6.98) | 0.191 | 0.14(-0.00, 0.29) | 0.058 |
| FACT-BMT | 6.65 (1.66, 11.64) | 0.009^b^ | 0.01 (-0.19, 0.20) | 0.944 | 3.27 (-2.74, 9.28) | 0.286 | 0.01 (-0.21, 0.24) | 0.917 |

^a^ Model adjusted for age, sex, education, body mass index (BMI), main caregivers, diagnosis, transplantation type, history of aGVHD and cGVHD, and history of infection.

^b^ indicates the statistical significance for the factors.

Abbreviations: GH, general health; PF, physical functioning; RP, role physical; RE, role emotional; SF, social functioning; BP, bodily pain; VT, vitality; MH, mental health; PWB, physical well-being; FWB, functional well-being; SWB, social well-being; EWB, emotional well-being, FACT-BMT, total with BMT module; TOI, FACT-BMT Trial Outcome Index (TOI).

**Additional file 1: TableS3.** Stratified analysis of QoL between HID and MSD patients in CMV and no-CMV reactivation groups.

|  | **No CMV Reactivation** | | | | **CMV Reactivation** | | | |
| --- | --- | --- | --- | --- | --- | --- | --- | --- |
| **QoL** | **HSCT (HID vs. MSD)** | | **Time (Months post-HSCT)** | | **HSCT (HID vs. MSD)** | | **Time (Months post-HSCT)** |  |
|  | **β (95% CI) ^a^** | ***P*** | **β (95% CI) ^a^** | ***P*** | **β (95% CI) ^a^** | ***P*** | **β (95% CI) ^a^** | ***P*** |
| SF-36 | | | | | | | | |
| GH | 6.46 (0.24, 12.69) | 0.042^b^ | 0.13 (-0.12, 0.39) | 0.305 | 4.54 (-5.01,14.11) | 0.351 | -0.03 (-0.31, 0.22) | 0.804 |
| PF | 6.02 (-1.22, 13.28) | 0.103 | 0.38 (0.09, 0.67) | 0.009^b^ | -7.22 (-20.77, 6.33) | 0.297 | 0.26 (-0.14, 0.65) | 0.200 |
| RP | 3.74 (-3.76, 11.25) | 0.329 | 0.97 (0.40, 1.53) | 0.001^b^ | 4.91(-9.04, 18.87) | 0.49 | 0.37 (-0.12, 0.86) | 0.135 |
| RE | 5.75 (-6.05, 17.55) | 0.340 | 0.52 (-0.09, 1.13) | 0.096 | -1.18 (-19.07, 16.71) | 0.897 | -0.10 (-0.69, 0.48) | 0.724 |
| SF | 6.83 (-1.30, 14.97) | 0.099 | 0.92 (0.54, 1.29) | 0.000^b^ | -1.22 (-13.19, 10.75) | 0.841 | 0.46 (0.08, 0.85) | 0.018^b^ |
| BP | 4.12 (-1.75, 9.99) | 0.169 | 0.18 (-0.05, 0.41) | 0.129 | 0.37 (-10.31, 11.05) | 0.946 | 0.10 (-0.24, 0.44) | 0.565 |
| VT | 8.69 (2.60, 14.78) | 0.005^b^ | 0.19 (-0.01, 0.39) | 0.064 | -2.12 (-10.76, 6.51) | 0.63 | 0.08 (-0.20, 0.36) | 0.582 |
| MH | 5.62 (0.25, 10.99) | 0.040^b^ | -0.04 (-0.20, 0.13) | 0.639 | 1.04 (-6.88, 8.96) | 0.797 | -0.08 (-0.34, 0.17) | 0.530 |
| FACT-BMT | | | | | | | | |
| PWB | 2.09 (0.33, 3.84) | 0.020^b^ | 0.12 (0.06,0.19) | 0.000^b^ | -0.88 (-3.34, 1.57) | 0.482 | 0.04 (-0.03, 0.11) | 0.227 |
| SWB | 1.66 (0.23, 3.09) | 0.023^b^ | -0.05 (-0.13, 0.04) | 0.314 | 2.04 (-0.07, 4.15) | 0.058 | -0.02 (-0.10, 0.05) | 0.497 |
| EWB | 0.97 (-0.38, 2.31) | 0.161 | -0.01 (-0.05, 0.03) | 0.644 | -0.79(-2.67, 1.08) | 0.406 | -0.00 (-0.05, 0.04) | 0.862 |
| FWB | 2.7 (1.01, 4.39) | 0.002^b^ | 0.01 (-0.10, 0.12) | 0.841 | -0.33 (-3.36, 2.70) | 0.83 | 0.05 (-0.06, 0.17) | 0.357 |
| FACT-G | 7.78 (3.33, 12.23) | 0.001^b^ | 0.04 (-0.19, 0.28) | 0.706 | -0.18 (-6.95, 6.59) | 0.958 | 0.08 (-0.11, 0.27) | 0.420 |
| TOI | 4.21 (1.29, 7.15) | 0.005^b^ | 0.07 (-0.07, 0.22) | 0.336 | -1.38 (-5.68, 2.91) | 0.529 | 0.10 (-0.02, 0.24) | 0.108 |
| FACT-BMT | 6.93 (2.44, 11.42) | 0.002^b^ | 0.01 (-0.22, 0.23) | 0.957 | -9.07 (-7.78, 5.96) | 0.796 | 0.08 (-0.11, 0.28) | 0.399 |

^a^ Model adjusted for age, sex, education, body mass index (BMI), main caregivers, diagnosis, transplantation type, history of aGVHD and cGVHD, and history of infection.

^b^ indicates the statistical significance for the factors.

Abbreviations: GH, general health; PF, physical functioning; RP, role physical; RE, role emotional; SF, social functioning; BP, bodily pain; VT, vitality; MH, mental health; PWB, physical well-being; FWB, functional well-being; SWB, social well-being; EWB, emotional well-being, FACT-BMT, total with BMT module; TOI, FACT-BMT Trial Outcome Index (TOI).
